# Matrix-state switching in osteosarcoma under therapeutic pressure: integrin rewiring, residual disease, and pulmonary niche adaptation

**DOI:** 10.3389/fcell.2026.1987843

**Published:** 2026-10-22

**Authors:** Yang Gu, Yunqiang Zhuang, Wei Zhang

**Affiliations:** 1 Trauma Orthopedics Center, Ningbo No.6 Hospital, Ningbo, Zhejiang, China; 2 Ningbo Clinical Research Center for Orthopedics, Sports Medicine & Rehabilitation, Ningbo, Zhejiang, China

**Keywords:** anoikis, extracellular matrix remodeling, integrin, lung metastasis, osteosarcoma, patient-derived models, therapy resistance, tumor ecosystem

## Abstract

Osteosarcoma cells encounter distinct extracellular environments during primary growth, treatment, detachment, and pulmonary colonization. A single constitutive extracellular-matrix (ECM)–integrin–FAK–AKT pathway therefore cannot explain residual disease and metastasis. This Review proposes matrix-state switching as a stage-resolved framework jointly defined by ligand presentation, adhesion-receptor usage, matrix-coupled signaling dependencies, and the cellular compartment sustaining these interactions. The framework is not an obligatory linear trajectory: observed state differences may reflect reversible adaptation, selection of pre-existing populations, therapy-associated remodeling, or combinations thereof. Primary lesions show component-selective matrix-conditioned drug tolerance; detached cells can preserve Src-dependent PI3K–AKT survival despite loss of sustained FAK and paxillin phosphorylation; and pulmonary colonization introduces distinct tumor-cell and stromal dependencies, including β4–ezrin-associated signaling and the CXCL14–α11β1 fibroblast circuit. Recent single-cell and spatial studies further indicate therapy-associated ECM remodeling and residual malignant-cell programs involving stemness, epithelial–mesenchymal transition, ECM interaction, and PI3K signaling, but do not establish same-clone longitudinal switching. LAMC1 is evaluated strictly as a hypothesis-generating matrix-state candidate because current osteosarcoma-specific evidence remains transcript-level. We position matrix-state switching as a matrix- and adhesion-focused layer within the broader communication-driven osteosarcoma ecosystem and define falsifiable criteria for distinguishing functional state dependencies from association.

## 1 Introduction

Osteosarcoma is often framed as a genetically heterogeneous malignancy, yet genetic diversity alone does not explain why a minority of cells survive matrix detachment, establish pulmonary metastases, and persist during multiagent chemotherapy. Each of these events occurs in a different extracellular setting. Primary tumor cells occupy a mineralizing, collagen-rich and fibronectin-rich environment. Disseminating cells temporarily lose productive anchorage and face anoikis. Cells that reach the lung must then regain adhesive competence in a matrix and stromal ecosystem unlike the bone lesion. Therapy resistance, in this setting, is not only a property of a tumor clone. It can emerge from the way that clone changes its relationship with the surrounding matrix. Contemporary osteosarcoma reviews likewise place metastatic failure, chemoresistance, and tumor-microenvironmental adaptation among the principal unresolved clinical problems (Isakoff et al., 2015; Smrke et al., 2021; Harris and Hawkins, 2022; Garcia-Ortega et al., 2022; Zhao et al., 2021; Cui et al., 2020; Yu et al., 2022; Rodrigues et al., 2022).

Historical tissue studies already suggested that osteosarcoma does not maintain one fixed ligand-receptor program. In 16 primary tumors, fibronectin and type-I collagen were broadly distributed, whereas β1-integrin alpha subunits were selective: α4 was detected in all tumors, α5 in 75%, and classical collagen- and laminin-binding receptors were uncommon (Kawaguchi and Uede, 1993). Every evaluable pulmonary metastasis differed from its paired primary lesion in at least part of the integrin repertoire, and two of three informative invasive fronts gained or strengthened integrin expression (Kawaguchi and Uede, 1993). Such observations are difficult to reconcile with a single constitutive ECM-integrin pathway.

Anoikis provides a more direct functional test. In parental SAOS-2 cells, suspension increased apoptosis from 10.3% to 51.8% and triggered mitochondrial cytochrome-c release and caspase-3 activation (Marco et al., 2003). The receptor effect was not uniform: one α4-directed antibody increased apoptosis in suspended cells, whereas the tested β1-directed antibodies did not reproduce that response (Marco et al., 2003). A suspension-selected resistant derivative maintained Src and AKT activation; Src or PI3K inhibition restored anoikis even though FAK and paxillin were dephosphorylated after detachment, and constitutively active FAK failed to rescue the sensitive parental cells (Diaz-Montero et al., 2006). The same downstream survival pathway can therefore persist while its upstream adhesive architecture changes.

Matrix context also modifies treatment response. In an early three-dimensional migration model, doxorubicin produced different antiproliferative and antimigratory effects in monolayer cells and cells recovered after migration into ECM gel; the migrated population showed higher β1 integrin, MMP9, and topoisomerase II (Harisi et al., 2005). Because drug exposure preceded the matrix overlay, this experiment cannot establish simultaneous matrix-mediated drug protection. It does show that treatment schedule, migration state, and matrix-associated phenotype are interdependent. Clinical material provides a related signal. In a retrospective series of 36 high-grade osteosarcomas, FAK expression was higher in poor histologic responders (P = 0.0212); an internally derived β5-FAK-GSK3β profile reached an AUC of 0.899, with 94% sensitivity, 86% specificity, and 90% accuracy, but lacked external validation (Le Guellec et al., 2013).

The metastatic lung introduces another receptor and another cellular compartment. Single-cell analysis identified a CXCL14-enriched osteosarcoma population with stem-like, sphere-forming, anoikis-resistant, tumor-initiating, and prometastatic features (Xu et al., 2024). Tumor-derived CXCL14 directly engaged α11β1 on fibroblasts, activating AKT/ERK signaling, focal-adhesion maturation, actomyosin contractility, matrix contraction, TGF-β production, and CD8-positive T-cell exclusion (Xu et al., 2024). The relevant mechanistic unit is therefore not always a tumor-cell-autonomous integrin pathway; a metastatic matrix state can be built through tumor-stromal communication.

LAMC1 is attractive in this context because laminin γ1 is shared by multiple laminin heterotrimers and could, in principle, connect matrix composition to receptor-dependent survival. The osteosarcoma evidence is not yet sufficient for that conclusion. Early pathology showed weak or absent laminin in most primary lesions (Kawaguchi and Uede, 1993), whereas Tang et al. reported a TARGET-derived survival association and heterogeneous LAMC1 mRNA expression across osteosarcoma cell lines: expression was higher than osteoblast controls in some lines but lower in others (Tang et al., 2022). The analysis lacked external or multivariable survival validation and did not establish patient-tumor γ1 protein, heterotrimer assembly, extracellular deposition, receptor engagement, or causal signaling. This evidentiary boundary is central to the argument developed below.

In this Review, a matrix state is operationally defined as a context-specific, experimentally distinguishable configuration jointly specified by extracellular ligand composition and presentation, adhesion-receptor usage and activation, matrix-coupled signaling dependencies, and the cellular compartment that sustains these interactions. Matrix-state switching refers to a reproducible redistribution between such configurations across disease or treatment contexts. It does not, by itself, imply longitudinal conversion of the same osteosarcoma clone, because reversible adaptation, selection of pre-existing subclones, and treatment-associated induction may coexist. The framework separates attached primary-tumor survival, detachment and anoikis escape, and pulmonary re-adhesion/niche construction while reserving “drug resistance” or “drug tolerance” for experiments that include a treatment challenge.

Matrix-state switching is not intended to replace ECM plasticity, integrin switching, mechanotransduction, or tumor–microenvironment communication. Rather, it integrates these processes at a stage-resolved level by treating the ligand–receptor–signaling–compartment configuration, and the context in which that configuration becomes functionally necessary, as the experimental unit. Its conceptual novelty therefore lies in linking state-specific extracellular organization to testable changes in receptor usage, signaling topology, and cellular compartment instead of inferring a single constitutive ECM–integrin pathway.

Targeted literature searches were performed primarily in PubMed through 2 September 2026, supplemented by reference-list screening of relevant primary studies and reviews. Search terms combined “osteosarcoma” with “extracellular matrix”, “integrin”, “anoikis”, “laminin”, “LAMC1”, “chemotherapy”, “drug resistance”, “lung metastasis”, “fibroblast”, “organoid”, “three-dimensional model”, and “salvianolic acid A”. Priority was given to osteosarcoma studies with functional perturbation, state-resolved models, paired clinical material, spatial or single-cell resolution, or direct target-deconvolution evidence. Cross-cancer studies were used only to define biological plausibility or experimental criteria. This is a focused narrative Review, not a systematic review, and the cited literature is illustrative rather than exhaustive. Representative stage-specific matrix modules and their evidence levels are summarized in Table 1.

**TABLE 1:** Stage-specific matrix modules and evidence levels in osteosarcoma. Studies were selected to represent distinct matrix states, cellular compartments, and evidence types. “Evidence type/level” describes study design rather than a numerical score; the table is illustrative rather than exhaustive and separates functional evidence from transcriptomic or descriptive association.

State/context and module	Study/model	Evidence type/level	Core finding	Interpretation	Key limitation
Primary/paired tissue — integrin repertoire remodeling	Kawaguchi and Uede, 1993; 16 primaries with paired metastatic/invasive material	Clinical descriptive/spatial pathology	Fibronectin and collagen I were common; integrin repertoires differed between primary and pulmonary lesions	Supports stage- and site-dependent receptor remodeling	Descriptive tissue data do not establish receptor dependency or same-clone switching
State I — ECM/HSPG–fibronectin protection after chemotherapy	Harisi et al., 2007; patient-derived OSCORT model after 4-h chemotherapy pulse	Patient-derived *in vitro* functional	Post-treatment ECM contact increased apparent drug tolerance; HSPG was most protective, fibronectin weaker, laminin/nidogen not protective	Demonstrates component-selective matrix-conditioned survival after treatment	Drug exposure preceded ECM overlay; specific receptor and p53-rescue causality were not established
State I — TGFBI–α2β1–PI3K module	Guo et al., 2014; SaOS-2/MG-63 perturbation plus tail-vein model	*In vitro* functional + *in vivo* supportive	TGFBI knockdown or α2/β1 blockade reduced adhesion/motility and AKT activation; TGFBI overexpression increased lung signal	Relatively complete ligand–receptor–signaling module	Tail-vein design cannot resolve the exact stage at which the module is required
State II — Src–PI3K–AKT anoikis-survival module	Diaz-Montero et al., 2006; suspension-selected SAOSar cells	*In vitro* functional perturbation	Src Tyr418 and AKT Ser473 persisted in resistant suspension; Src/PI3K inhibition restored anoikis while FAK/paxillin remained low	Defines a FAK-independent detached-state survival topology	Selected-cell model and pharmacological specificity limit generalization
State III, tumor-cell intrinsic — β4–ezrin/AKT–S6	Wan et al., 2009; β4 perturbation and experimental lung colonization	*In vivo* functional	β4 knockdown or Tyr1494 disruption strongly reduced lung colonization; β4 associated with ezrin and AKT/S6 signaling	Supports a metastasis-selective tumor-cell adhesion dependency	Tail-vein model bypasses early dissemination; β4 re-expression suggests escape selection
State III, stromal-mediated — CXCL14→fibroblast α11β1	Xu et al., 2024; scRNA-seq, fibroblast assays, mice and PDX	Multimodal functional/compartment-resolved	Tumor-derived CXCL14 engaged fibroblast α11β1, activated AKT/ERK, remodeled matrix and TGF-β output; blockade suppressed lung metastasis	Strong tumor–stroma niche mechanism; α11β1 belongs principally to fibroblasts in this model	Does not represent tumor-cell-intrinsic integrin switching and remains model-specific
Therapy-associated ecosystem remodeling	Zheng et al., 2024b; Hu et al., 2026; single-cell analyses before/after chemotherapy	Paired/temporal transcriptomic association	Post-chemotherapy samples show stromal remodeling and residual malignant-cell programs involving ECM, EMT and PI3K-related signaling	Supports therapy-associated state redistribution	Cannot distinguish treatment-induced state change from selection/enrichment of pre-existing populations without lineage tracing
LAMC1 candidate	Tang et al., 2022; TARGET-derived association and cell-line qRT-PCR	Transcriptomic hypothesis-generating	LAMC1 mRNA was heterogeneous; a survival association was reported	Useful as an evidence-ladder example, not an established driver or matrix dependency	γ1 protein, heterotrimer assembly, deposition, receptor engagement, state-specific signaling and rescue remain untested

## 2 Matrix-state switching as a stage-resolved framework

### 2.1 Four layers define a matrix state

A matrix state is more than the abundance of an ECM molecule. Four linked layers determine whether a particular extracellular environment is biologically competent. The ligand layer includes abundance, isoform composition, heterotrimer assembly, extracellular deposition, proteolysis, and mechanical presentation. The receptor layer includes the integrin repertoire, activation and clustering, ligand specificity, and the cell type in which the receptor is expressed. The signaling layer encompasses FAK-dependent and FAK-independent routes converging on Src, PI3K-AKT, ERK, Rho-ROCK-MLC2, mitochondrial apoptosis, and TGF-β. The ecosystem layer identifies the participating compartments: attached primary tumor cells, detached or spheroid-like cells, fibroblasts, immune cells, and pulmonary lesions. This formulation is consistent with broader matrisome, integrin, and mechanotransduction literature in which biochemical composition, force transmission, matrix stiffness, stromal remodeling, and receptor state jointly determine cancer-cell behavior (Zhao et al., 2025; Hynes and Naba, 2012; Mouw et al., 2014; Bonnans et al., 2014; Kechagia et al., 2019; Hamidi and Ivaska, 2018; Desgrosellier and Cheresh, 2010; Bergonzini et al., 2022; Jiang et al., 2022; Yuan et al., 2023; Su et al., 2020).

These layers are related but not interchangeable. A ligand can be abundant while its cognate receptor is scarce. A receptor can remain detectable while its activation state changes. AKT phosphorylation can recur at several disease stages even when the upstream receptor and adaptor architecture is different. A bulk RNA signal may derive from tumor cells, fibroblasts, or another stromal population and may not correspond to a deposited matrix structure. Matrix-state switching refers to changes in functional coupling among these layers, rather than to expression changes considered in isolation.

Operationally, evidence for a distinct matrix state requires more than differential expression: coordinated differences should be demonstrated in extracellular ligand or deposition, receptor engagement, signaling architecture, and a state-relevant phenotype. Evidence for true same-lineage switching is more stringent and additionally requires lineage-resolved transition between states, ideally with state-specific perturbation and rescue. No existing osteosarcoma study has yet followed the same clone through all three contexts while satisfying these criteria. The framework is therefore a falsifiable synthesis of state redistribution, not a claim that longitudinal switching has already been directly observed. Circulating tumor-cell clusters, platelet or fibrin shielding, and dormancy may also provide matrix-bearing or matrix-independent alternatives to a strictly matrix-free circulating state.

### 2.2 Three selective states organize the available osteosarcoma evidence

The existing evidence can be arranged around three experimentally distinguishable contexts. State I is the attached primary tumor, where cells interact with a mineralized, collagen- and fibronectin-rich matrix and may acquire matrix-conditioned drug tolerance. State II captures the detachment/anoikis bottleneck, in which conventional focal-adhesion signaling is disrupted and survival requires tolerance of anchorage loss. State III represents pulmonary re-adhesion and niche construction, where surviving populations establish productive tumor-cell or stromal matrix interactions in the lung. These states organize evidence; they are not intended to prescribe a single obligatory metastatic route.

The framework explicitly allows branching trajectories. Cells may enter dormancy, travel as matrix-bearing clusters, acquire platelet/fibrin shielding, or reach a pulmonary niche after selection of pre-existing dissemination-competent subclones. Apparent transitions may therefore reflect reversible adaptation, population selection, or both. The purpose of the three-state organization is to ask whether a pathway is required in the context in which it is being interpreted. FAK-associated expression may correlate with treatment response in a primary biopsy (Le Guellec et al., 2013), while FAK is dispensable for the Src–AKT survival program selected under suspension (Diaz-Montero et al., 2006). AKT is again activated in fibroblasts downstream of CXCL14–α11β1 during metastatic niche formation (Xu et al., 2024). These are not one continuous pathway.

Figure 1 summarizes the proposed matrix-state framework as a branching, non-obligatory process. The labeled receptors are model-specific examples rather than universally dominant integrins; α11β1 is assigned specifically to the fibroblast compartment. Side paths and annotations indicate alternative trajectories such as dormancy, matrix-bearing clusters, or platelet/fibrin shielding, and the diagram does not establish that a single clone completes every transition.

**FIGURE 1:**
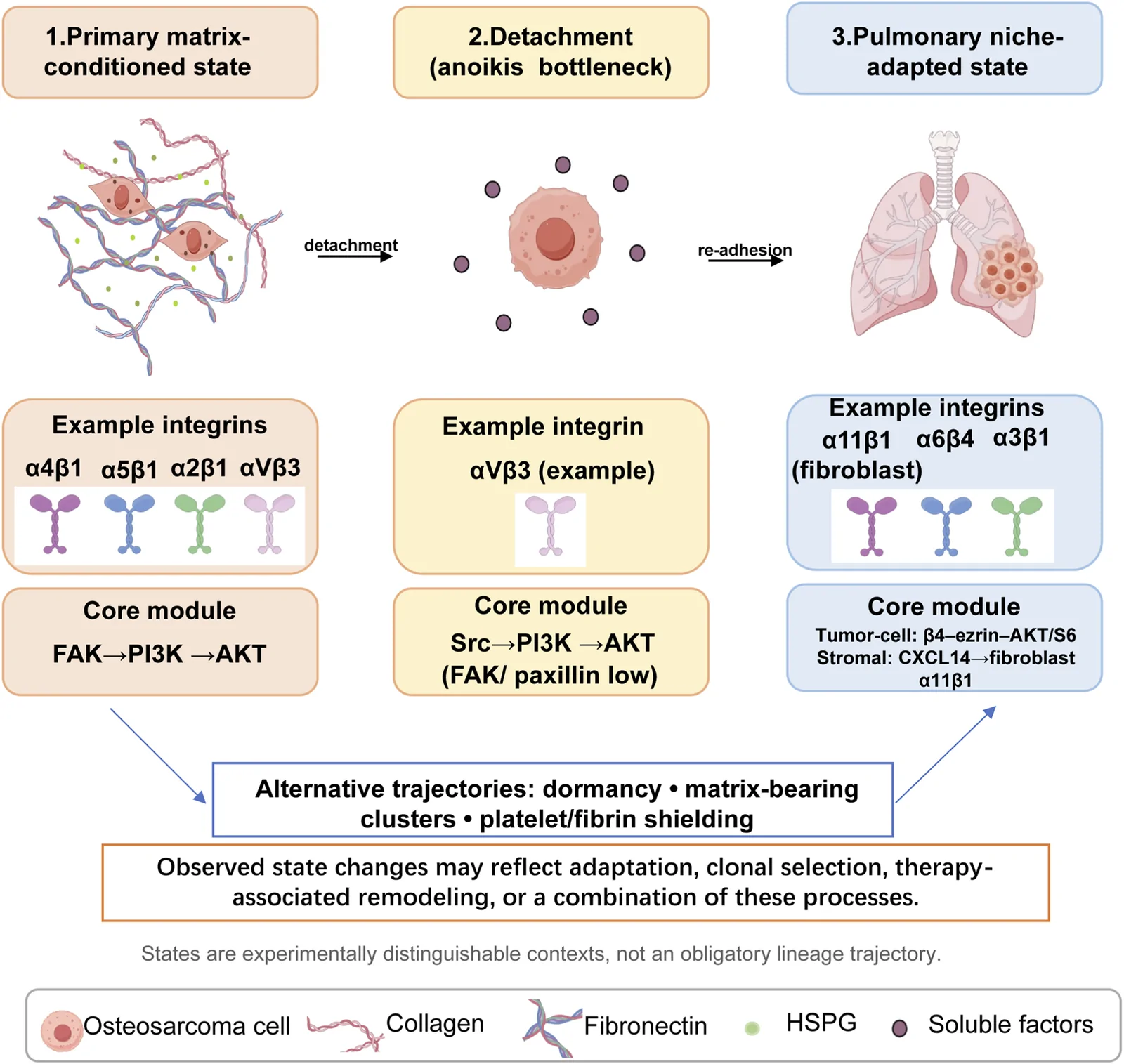
Matrix-state framework across osteosarcoma progression as a branching, non-obligatory process. The diagram separates a primary matrix-conditioned state, a detachment/anoikis bottleneck, and a pulmonary niche-adapted state while explicitly allowing alternative trajectories such as dormancy, matrix-bearing clusters, and platelet/fibrin shielding. Observed state redistribution may reflect reversible adaptation, selection of pre-existing subclones, therapy-associated remodeling, or combinations thereof. In State III, β4–ezrin-associated signaling is shown as a tumor-cell module, whereas CXCL14–α11β1 is assigned to fibroblasts. The scheme is an operational hypothesis and does not demonstrate that one clone completes every transition.

### 2.3 Matrix-state switching within a communication-driven osteosarcoma ecosystem

A recent communication-driven model conceptualizes osteosarcoma as an emergent multicellular ecosystem sustained by reciprocal signaling among malignant, stromal, immune, endothelial, bone-resident, and metastatic-niche compartments (Ricciardi et al., 2026). Matrix-state switching is complementary rather than competing with this framework. The communication-driven model defines the broader intercellular information network, whereas matrix-state switching focuses on one experimentally tractable layer within that network: stage-specific coupling of extracellular ligand presentation, adhesion-receptor usage, matrix-responsive signaling, and the cellular compartment that sustains these interactions. Tumor–stroma communication can therefore generate or stabilize a matrix state, while a matrix-state redistribution can in turn alter the communication network.

### 2.4 The therapeutic unit is the transition, not the pathway name

A state-transition model changes the therapeutic question. Blocking a molecule that is important in one state may have little effect in the next, and receptor substitution can preserve a downstream survival output even when one adhesion module is lost. Conversely, a short-lived transition may create a vulnerability that is invisible in stable monolayer culture. The most informative targets are therefore those that are nonredundant during a transition, or those that prevent cells from rebuilding a viable signaling architecture after the transition has occurred. Work on ECM-associated stemness, integrin-dependent drug resistance, focal-adhesion plasticity, and anoikis resistance supports the same need to resolve when a dependency becomes operative rather than treating pathway expression as a fixed property (Kesh et al., 2020; Liu et al., 2025; Kariya and Nishita, 2025; Shaw et al., 2025; Zhao et al., 2025).

Figure 2 converts LAMC1 into an evidence-ladder example rather than a proposed driver pathway. In osteosarcoma, only transcript-level association has been reported; laminin assembly, extracellular deposition, receptor engagement, state-specific signaling, phenotype, and rescue remain experimental requirements.

**FIGURE 2:**
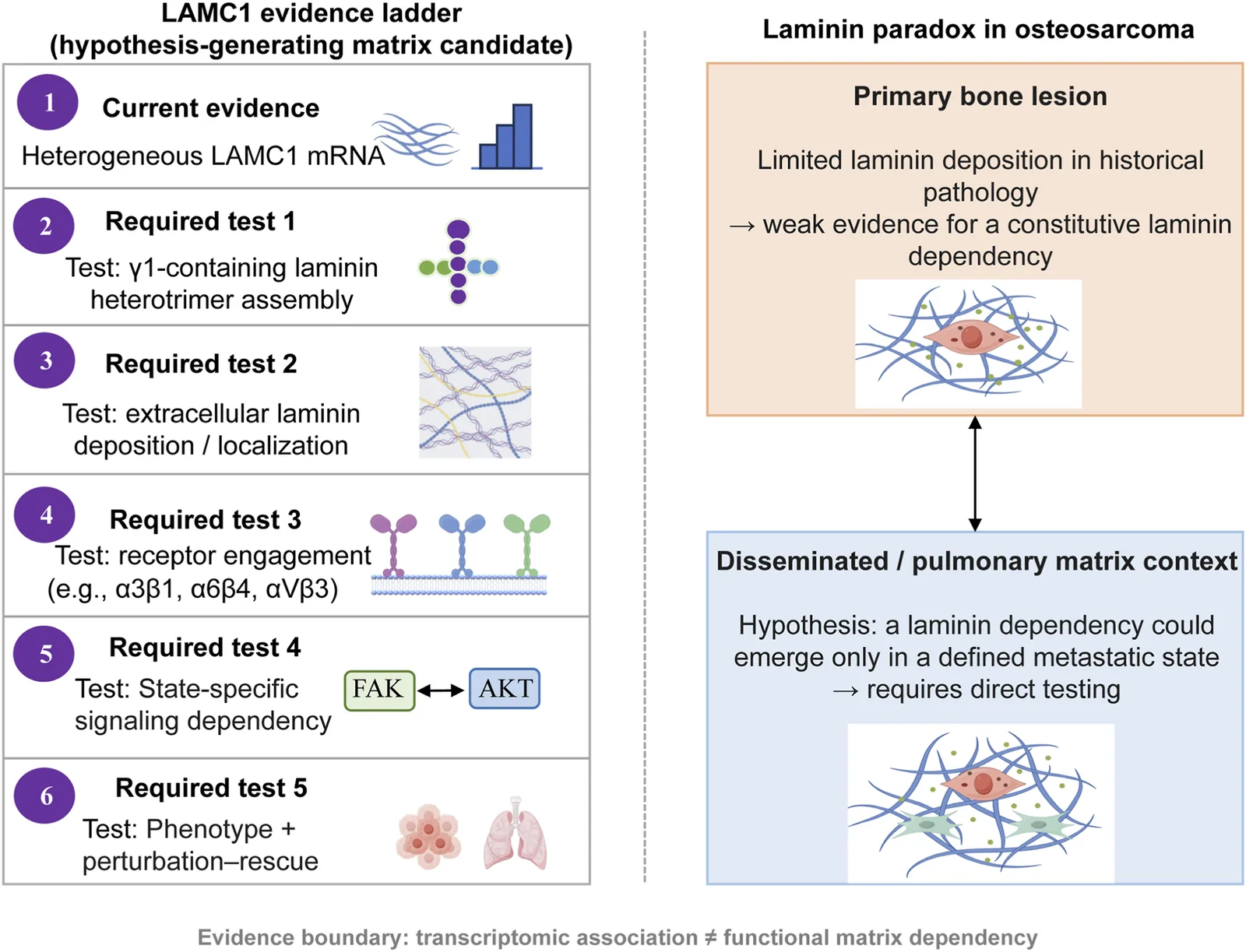
LAMC1 as a hypothesis-generating matrix candidate: an evidence ladder. Current osteosarcoma-specific support is limited to heterogeneous cell-line mRNA expression and a TARGET-derived survival association. γ1-containing laminin assembly, extracellular deposition, defined receptor engagement, state-specific signaling, phenotype, and perturbation–rescue remain required tests rather than established steps. The figure is evidentiary, not a proposed linear LAMC1 pathway.

### 2.5 Therapy-associated versus progression-associated matrix remodeling

Most historical studies reviewed below model progression-associated adaptation or compare stable experimental states; they do not show that chemotherapy caused the observed matrix program. The strongest human evidence is therefore described as therapy-associated remodeling or post-chemotherapy enrichment. Such changes may reflect treatment-induced remodeling, selective survival/enrichment of pre-existing resistant populations, or both, and should not be labeled “chemotherapy-induced matrix switching” without lineage-resolved temporal evidence (Zheng et al., 2024b).

A 2026 matched analysis of three patients before and after neoadjuvant chemotherapy likewise found expansion of fibroblast and endothelial compartments, contraction of immune populations, and residual malignant-cell programs involving epithelial–mesenchymal transition, ECM interaction, and integrin/PI3K-related signaling (Hu et al., 2026). Its paired design strengthens temporal inference, but the small cohort, transcript-level readouts, pseudotime reconstruction, and computational drug-sensitivity predictions do not establish that chemotherapy directly selects a specific ECM–integrin dependency. Together, these studies support therapy-associated ecosystem remodeling and residual-state adaptation; prospective lineage tracing, spatial protein validation, and perturbation are still required to distinguish induction from selection of pre-existing states.

## 3 Primary-tumor adhesion and matrix-conditioned resistance

### 3.1 Matrix protection is component-selective and temporally gated

The primary osteosarcoma matrix should not be treated as a uniform physical barrier. Fibronectin and type-I collagen were broadly detected in tumor tissue, but the corresponding receptor repertoire was uneven (Kawaguchi and Uede, 1993). This mismatch suggests that signaling competence depends on the combination of ligand identity, receptor availability, receptor state, and the timing of matrix contact relative to treatment. More recent osteosarcoma studies extend this heterogeneity: fibronectin/αvβ3 expression has been associated with chemotherapy response and outcome; collagen I can signal through α2β1-JAK-STAT3; LOX can increase FAK phosphorylation; and Tspan7 or Tspan9 can couple β1 integrin to FAK-Src/Ras-ERK signaling (Shi et al., 2019; Wei et al., 2020; Chen et al., 2023; Shao et al., 2022a; Shao et al., 2022b).

A patient-derived OSCORT model provides direct evidence that post-treatment matrix contact can modify tumor-cell fate (Harisi et al., 2007). Cells were exposed for 4 h on plastic to methotrexate, cisplatin, maphosphamide, or doxorubicin and were then maintained either on plastic or beneath an EHS-derived ECM gel. Apparent IC50 values were higher after transfer into ECM for all four agents, with the largest difference after doxorubicin, although numerical IC50 values were not reported (Harisi et al., 2007). Since drug exposure occurred before ECM overlay, reduced drug penetration cannot fully explain the subsequent difference in survival.

The DNA-damage phenotype was especially clear. On plastic, DNA fragmentation reached 244% and 238% of untreated control after 0.5 and 5 μg/mL doxorubicin, respectively; neither concentration produced a significant increase above the corresponding ECM control (Harisi et al., 2007). Comet-tail measurements likewise showed more frequent and more extensive damage on plastic. ECM exposure reduced total p53 protein and prevented efficient nuclear accumulation after doxorubicin. Common p53 mutations were not detected, leading the authors to propose an epigenetic loss of p53 function (Harisi et al., 2007). The study did not perform p53 re-expression or nuclear-targeting rescue, so altered DNA repair, cell-cycle state, or apoptosis threshold cannot be excluded.

The protective phenotype was not shared by all ECM constituents. Heparan sulfate proteoglycan produced the strongest protection and fibronectin a weaker effect. Laminin and nidogen did not reduce doxorubicin cytoreduction, whereas collagen IV increased it (Harisi et al., 2007). This result places an important boundary around any future LAMC1 hypothesis: greater laminin abundance cannot be assumed to be protective without defining the assembled laminin, receptor context, and disease state.

### 3.2 Soluble matrix-associated proteins can convert inflammatory stress into survival

Matrix-conditioned survival can also be mediated by extracellular proteins that are not structural scaffolds. In MG-63 cells, TNF-α alone, even at 100 ng/mL, produced little death, whereas cycloheximide sensitization enabled caspase-3 activation and PARP cleavage (Tsagaraki et al., 2010). Under the resistant condition, TNF-α increased TIMP1 secretion and cell-surface αvβ3 while decreasing β1 integrin. TIMP1 knockdown reduced Bcl-xL by approximately 50% and increased TNF-α-induced PARP cleavage. Recombinant TIMP1 at 800 ng/mL reduced apoptosis by approximately 37%, reduced PARP cleavage by approximately 35%, and restored Bcl-xL (Tsagaraki et al., 2010).

Co-immunoprecipitation and surface confocal microscopy supported an association between TIMP1 and αvβ3. Echistatin, used to disrupt αvβ3 ligation, reduced the anti-apoptotic effect of recombinant TIMP1 by approximately 30% (Tsagaraki et al., 2010). The evidence supports a TIMP1-αvβ3 survival module, but its boundaries are narrow. The system modeled TNF-α plus protein-synthesis inhibition rather than chemotherapy or detachment; echistatin was not a heterodimer-specific genetic perturbation; and although TNF-α activated AKT, p38, JNK, and NF-κB-related signaling, the study did not establish that the anti-apoptotic effect of TIMP1-αvβ3 itself required AKT.

### 3.3 TGFBI provides a ligand-receptor-PI3K bridge

A more complete ligand-receptor signaling chain was demonstrated for TGFBI, also known as βig-h3 (Guo et al., 2014). TGFBI knockdown reduced adhesion by 57.6% and 52.3%, invasion by 37.1% and 31.2%, and migration by 39.2% and 45.4% in SaOS-2 and MG-63 cells, respectively (Guo et al., 2014). Endogenous TGFBI co-localized and co-immunoprecipitated with α2 and β1 integrin subunits without changing their abundance. Blocking either subunit phenocopied TGFBI loss, and combined ligand knockdown plus receptor blockade produced no further reduction, consistent with a shared pathway. The second FAS1 domain reproduced the adhesion, invasion, and migration effects of full-length TGFBI (Guo et al., 2014).

TGFBI depletion lowered AKT phosphorylation. Blocking α2 or β1, or treating cells with the PI3K inhibitor LY294002, reduced AKT activation and motility phenotypes (Guo et al., 2014). Tail-vein delivery of TGFBI-overexpressing SaOS-2 cells increased lung fluorescence, supporting a prometastatic role *in vivo*. The model begins after escape from the primary tumor, however, and cannot separate effects on circulation survival, lung arrest, extravasation, and outgrowth. TGFBI-α2β1 is therefore a well-supported ligand-receptor-PI3K module whose stage specificity remains unresolved.

### 3.4 FAK-associated clinical signals require external validation

The 36-patient biopsy study adds clinical relevance without establishing a biomarker (Le Guellec et al., 2013). FAK expression was higher in poor responders to neoadjuvant chemotherapy (P = 0.0212). The internally constructed β5-FAK-GSK3β profile achieved an AUC of 0.899, 94% sensitivity, 86% specificity, and 90% accuracy (Le Guellec et al., 2013). These values are hypothesis-generating rather than clinically deployable because of the small cohort, multiple-marker modeling, and lack of an external validation set. They do, however, fit the broader observation that attached-state adhesion signaling can intersect with treatment response.

### 3.5 Primary-tumor resistance is a collection of non-equivalent modules

The attached state contains several distinct survival architectures. HSPG and fibronectin can attenuate doxorubicin-associated damage (Harisi et al., 2007); TIMP1 is associated with αvβ3-dependent buffering of TNF-α/cycloheximide-induced apoptosis (Tsagaraki et al., 2010); TGFBI can couple α2β1 to PI3K-AKT-dependent motility (Guo et al., 2014); and FAK-centered expression is clinically associated with histologic response (Le Guellec et al., 2013). None of these findings justifies a universal ECM-integrin-FAK-AKT pathway. Matrix composition, receptor activation, and exposure sequence have to be measured in the same experimental window as the phenotype being attributed to them. Related osteosarcoma data connect FAK abundance, αvβ3/αvβ5 dependency, CD44, PAI-1/MMP13, and the MMP2/MMP9 balance to metastatic or treatment-associated phenotypes, while biomimetic 3D matrices can expose drug responses that differ from two-dimensional culture (Gu and Zhou, 2018; Gvozdenovic et al., 2016; Gao et al., 2015; Liu et al., 2018; Hirahata et al., 2016; Kunz et al., 2016; Pavlou et al., 2019; Rao et al., 2025).

## 4 Detachment, anoikis escape, and receptor rewiring

### 4.1 Detachment selects a signaling topology distinct from focal adhesion

Detachment is not simply a weaker version of the attached state. In parental SAOS-2 cells, suspension increased apoptosis from 10.3% to 51.8% and engaged mitochondrial cytochrome-c release and caspase-3 (Marco et al., 2003). Repeated suspension selection generated an anoikis-resistant derivative in which Src Tyr418 and AKT Ser473 remained active (Diaz-Montero et al., 2006). Pharmacological inhibition of either Src or PI3K restored anoikis. FAK and paxillin were dephosphorylated after detachment in both sensitive and resistant cells, and constitutively active FAK did not rescue the parental phenotype (Diaz-Montero et al., 2006).

Src activity was upstream of AKT in the resistant suspended state. Treatment with 50 μM PP2 reduced AKT Ser473 phosphorylation, whereas the inactive control PP3 did not (Diaz-Montero et al., 2006). PTEN expression and activity did not differ in a way that explained the resistant phenotype. The data therefore define a detached-state Src-PI3K-AKT program that can maintain survival without persistent focal-adhesion signaling. Subsequent osteosarcoma studies broaden the detached-state network without replacing this Src-AKT core: RanBP9/TSSC3 suppresses Src-dependent AKT signaling, whereas FASN-ERK/Bcl-xL, ID1-PI3K-AKT, and FOSL1-SOX2 programs have each been linked to anoikis resistance or metastatic fitness; anoikis-resistant cells can also increase Src-MAPK-dependent angiogenic signaling (Dai et al., 2016; Gao et al., 2019; Sun et al., 2019; Zhao et al., 2019; Wang et al., 2024).

### 4.2 α4 illustrates why receptor abundance does not predict receptor function

The α4-integrin findings sharpen this point. One α4-directed antibody increased apoptosis of suspended SAOS-2 cells by 52%, while the tested β1 antibodies did not reproduce the effect (Marco et al., 2003). Because the phenotype depended on the epitope targeted and was not supported by α4 knockout or ligand-defined rescue, the result cannot be generalized as proof that α4 is intrinsically pro-anoikis. Integrin function depends on conformation, ligand occupancy, clustering, adaptor recruitment, and mechanical context. A receptor can be present in two states yet transmit different outcomes.

### 4.3 Loss of adhesion can coexist with increased metastatic fitness

Caveolin-1 perturbation exposes an adhesion-survival paradox (Cantiani et al., 2007). Osteosarcoma samples expressed less caveolin-1 than normal osteoblasts. In U-2 OS cells, caveolin-1 loss reduced adhesion to collagen I, collagen IV, fibronectin, and laminin by 50%–80%, but increased soft-agar growth, survival on poly-HEMA, migration, invasion, and experimental lung colonization (Cantiani et al., 2007). Mice receiving caveolin-1-deficient cells developed more than 250 lung metastases, compared with a median of two after parental cells and none after caveolin-1-overexpressing cells. Caveolin-1 gain or loss did not materially change monolayer proliferation or doxorubicin sensitivity (Cantiani et al., 2007).

Mechanistically, caveolin-1 constrained c-Src and Met signaling. Caveolin-1-deficient cells showed increased Src abundance, Tyr416 phosphorylation, and enzymatic activity; PP2 suppressed their migration, whereas the Src-activating peptide pYEEI restored motility to caveolin-1-overexpressing cells (Cantiani et al., 2007). Caveolin-1 also co-localized with Met and attenuated HGF-induced Src and AKT signaling. In 37 tumors, higher caveolin-1 was associated with better overall survival but not event-free survival (Cantiani et al., 2007). Reduced static adhesion therefore does not necessarily reduce metastatic fitness when survival and motility signaling is simultaneously rewired.

### 4.4 Detachment should be interpreted as a transient bottleneck

The detached state is experimentally attractive because it reveals a dependency that is less obvious during stable adhesion. Yet a disseminating cell does not remain matrix-free indefinitely. Anoikis escape is one bottleneck in a sequence. Surviving cells must later arrest, re-adhere, and rebuild signaling in the lung. A drug that is highly active during suspension could fail after re-adhesion, while an adhesion-targeted drug might show little effect during the interval in which FAK-dependent signaling is already lost. State-specific timing is therefore part of the mechanism rather than a secondary pharmacological detail.

## 5 Pulmonary re-adhesion and metastatic niche construction

### 5.1 Re-adhesion is a second selective bottleneck

Survival after detachment is necessary for metastasis but is not sufficient. Osteosarcoma cells that tolerate anchorage loss must subsequently interact with a pulmonary matrix that differs from bone and establish the adhesive signaling required for persistent growth. Historical paired-tissue observations are consistent with this transition: all evaluable pulmonary metastases altered at least part of their integrin repertoire relative to the matched primary tumor (Kawaguchi and Uede, 1993). α6, weak in most primary lesions, appeared or strengthened in selected invasive or metastatic regions (Kawaguchi and Uede, 1993).

The functional implication is that metastatic competence may require reacquisition of receptor programs suited to the lung rather than simple preservation of the primary-tumor receptor state. Standard monolayer proliferation assays are poorly designed to detect this type of dependency.

### 5.2 β4-ezrin signaling illustrates receptor reassembly in the lung

β4 integrin provides the clearest osteosarcoma example of a metastasis-selective adhesion module (Wan et al., 2009). β4 was strongly expressed in 45 of 48 human tumors, a near-uniform pattern that limited its value as a simple prognostic discriminator. In highly metastatic MNNG-HOS cells, β4 knockdown did not alter monolayer proliferation and produced inconsistent effects in routine migration or invasion assays, yet anchorage-independent growth and experimental pulmonary colonization were markedly reduced (Wan et al., 2009). Fifty days after tail-vein injection, pulmonary bioluminescence was detected in all eight control animals, compared with one of eight and two of nine mice receiving two independent β4-knockdown populations. All controls died before day 106, whereas approximately 70% of animals carrying β4-deficient cells remained alive at day 125 (Wan et al., 2009).

The long β4 cytoplasmic tail contains Tyr1494, which contributes to PI3K signaling. A Y1494F dominant-negative construct reduced β4 tyrosine phosphorylation, AKT and S6 phosphorylation, experimental lung metastasis, and mortality, while affecting primary intramuscular tumor growth much less (Wan et al., 2009). β4 also associated with ezrin. Loss or functional disruption of ezrin reduced β4 RNA and protein, whereas β4 depletion did not comparably reduce ezrin, suggesting that ezrin helps organize or stabilize a β4-dependent metastatic state. β4 expression re-emerged in late lesions after initial knockdown, consistent with selection for escape variants or receptor recovery (Wan et al., 2009).

These data also clarify why recurrent AKT activation should not be read as one continuous pathway. During suspension, Src-dependent PI3K-AKT survives despite loss of FAK (Diaz-Montero et al., 2006). During pulmonary colonization, β4, Tyr1494, and ezrin are components of another upstream architecture (Wan et al., 2009). The downstream kinase is shared; the causal network is not.

### 5.3 Tumor cells can actively construct a permissive pulmonary matrix state

Pulmonary re-adhesion is not exclusively tumor-cell autonomous. Xu and colleagues combined single-cell profiling of eight treatment-naive biopsies (23,798 cells) with analyses of primary and lung metastatic tissues, functional fibroblast studies, mouse experiments, and a PDX model (Xu et al., 2024). A CXCL14-high osteosarcoma population showed stem-like and anoikis-resistant properties. Secreted CXCL14 bound fibroblast α11β1 with a reported dissociation constant of 25.7 nM and activated AKT/ERK signaling, focal-adhesion maturation, actomyosin contractility, and matrix contraction (Xu et al., 2024). Independent studies now place this process within a wider premetastatic-niche program: osteosarcoma secretomes can activate resident fibroblasts and promote fibronectin/collagen remodeling, while tumor-derived extracellular vesicles can reprogram lung fibroblasts or immune populations before overt metastatic growth (Almeida et al., 2023; Mazumdar et al., 2020; Deng et al., 2024; Palmer et al., 2025; Li et al., 2026).

CXCL14-stimulated fibroblasts produced TGF-β and promoted osteosarcoma invasion and migration. Antibodies targeting the CXCL14-α11β1 axis reduced fibroblast TGF-β production, improved CD8-positive T-cell-mediated antitumor activity, and suppressed lung metastasis (Xu et al., 2024). The operative unit is therefore multicellular: tumor-derived CXCL14 activates a stromal integrin, which remodels mechanics and cytokine signaling while changing immune accessibility.

The compartment assignment is mechanistically essential. Bulk expression of ITGA11 or ITGB1 cannot establish a tumor-cell-intrinsic receptor state. In the CXCL14 model, α11β1 is principally a fibroblast receptor, so this axis represents stromal-mediated matrix adaptation rather than malignant-cell integrin switching. Spatial and single-cell resolution are therefore required to distinguish tumor-cell-intrinsic matrix rewiring from stromal mechanisms that construct or stabilize a permissive niche.

### 5.4 Matrix mechanics and immune exclusion expand the meaning of resistance

Primary-site ECM can modify chemotherapy-associated DNA damage (Harisi et al., 2007). In the lung, matrix remodeling can instead alter contractility, stromal signaling, and immune access (Xu et al., 2024). These observations link matrix remodeling to metastatic fitness and therapeutic context, but neither metastatic colonization nor immune exclusion is equivalent to demonstrated resistance to a specified treatment. In the reported model, the CXCL14–α11β1 circuit linked fibroblast focal-adhesion maturation and actomyosin tension to TGF-β production, matrix remodeling, and reduced CD8-positive T-cell access. It does not establish resistance to a specific osteosarcoma immunotherapy regimen, but it demonstrates that a metastatic matrix program can influence a determinant of therapeutic responsiveness beyond drug penetration. Recent syntheses and time-resolved studies further describe EV-mediated premetastatic niches and wound-healing/fibrotic programs that facilitate pulmonary colonization, reinforcing the view that metastatic resistance is partly an ecosystem phenotype (Xia et al., 2024; Reinecke et al., 2025a; Reinecke et al., 2025b; Bashir et al., 2025).

Matrix-directed therapy therefore need not mean nonspecific ECM depletion. The intervention point may be a tumor-derived ligand, a stromal integrin, mechanotransduction, or a downstream cytokine circuit. Re-adhesion and niche construction constitute a second therapeutic window after anoikis escape.

Figure 3 juxtaposes a tumor-cell-intrinsic β4–ezrin-associated pulmonary-colonization mechanism with a stromal-mediated CXCL14–fibroblast α11β1 circuit. The β4 study supports β4-subunit function, β4–ezrin association, and Tyr1494-dependent AKT/S6 signaling but did not identify a laminin ligand or prove a linear β4→ezrin pathway. The CXCL14 arm places α11β1 in fibroblasts. The two mechanisms have not been shown to interact directly or to depend on LAMC1.

**FIGURE 3:**
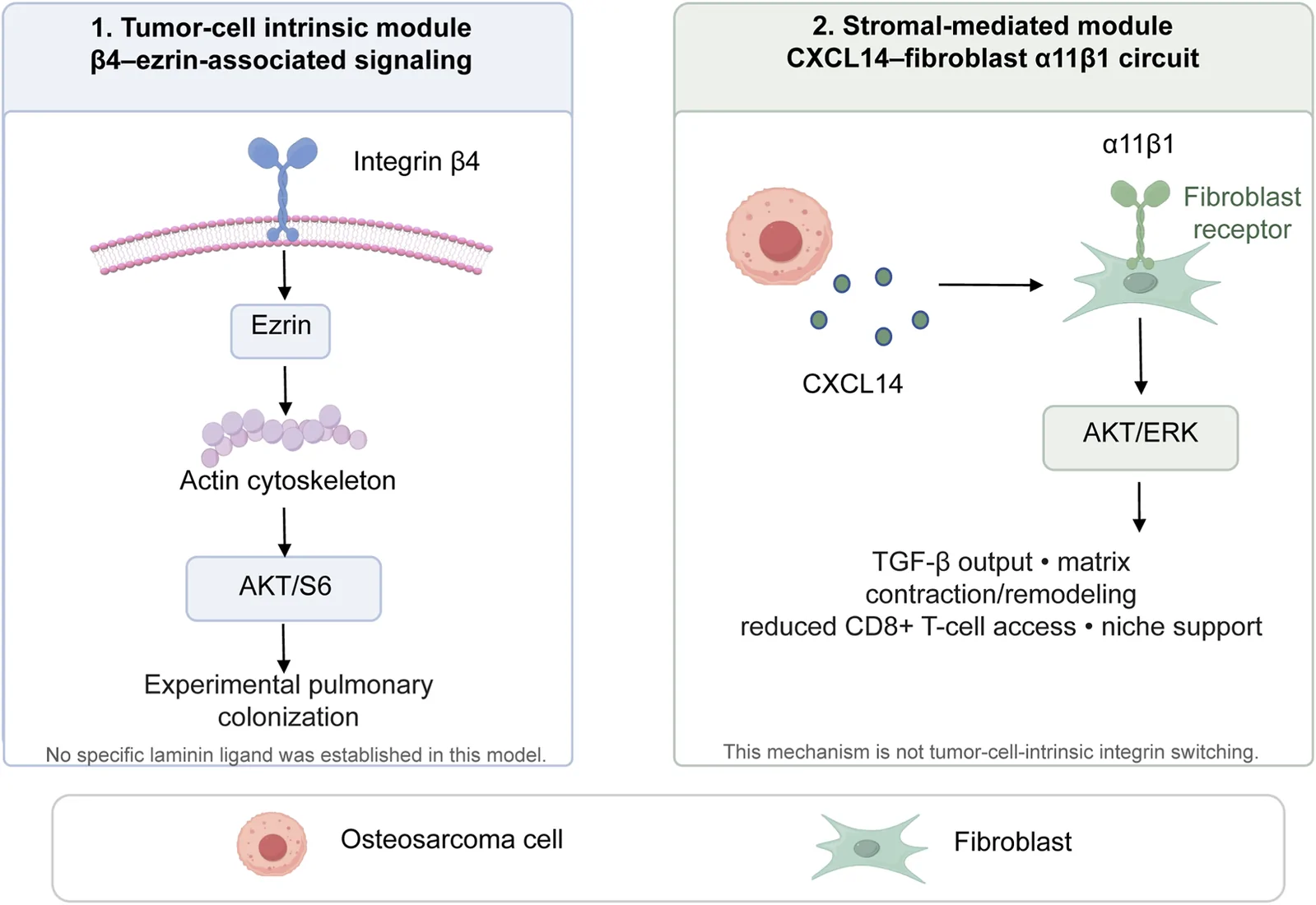
Distinct tumor-intrinsic and stromal-mediated pulmonary-colonization mechanisms. The tumor-cell model summarizes β4-subunit function, β4–ezrin association, and β4 Tyr1494-dependent AKT/S6 signaling (Wan et al., 2009); it does not establish a specific laminin ligand or a simple linear β4→ezrin cascade. The stromal model summarizes tumor-derived CXCL14 engagement of fibroblast α11β1 and associated AKT/ERK, matrix-remodeling, TGF-β, and immune-access phenotypes (Xu et al., 2024). The mechanisms arise from separate models and have not been shown to connect directly or depend on LAMC1.

## 6 The laminin paradox and LAMC1: from transcriptomic signal to functional matrix dependency

### 6.1 Osteosarcoma-specific evidence and limitations

LAMC1 is used here strictly as a hypothesis-generating case study for the matrix-state framework, not as an established osteosarcoma driver, biomarker, or therapeutic target. Tang et al. (2022) identified LAMC1 in a prognostically associated regulatory network, reported heterogeneous qRT-PCR expression across osteosarcoma cell lines, and described a TARGET-derived survival association. The study did not establish γ1 protein abundance, laminin heterotrimer assembly, extracellular deposition, receptor engagement, state-specific signaling, or a perturbation–rescue phenotype. The prognostic result was not externally or multivariably validated. Accordingly, current osteosarcoma-specific support stops at transcript-level association.

### 6.2 Laminin paradox and evidence ladder

Historical pathology reported only faint laminin staining in five of 16 primary tumors and weak α6-subunit staining in four of 16 (Kawaguchi and Uede, 1993). This does not contradict a transcriptomic LAMC1 signal because transcript abundance, γ1 protein, heterotrimer assembly, extracellular deposition, and receptor competence are distinct biological layers. It instead creates a testable paradox: a laminin dependency might be absent from much of the primary tumor yet emerge in a disseminated or pulmonary matrix context. Cross-cancer studies support the general plausibility of LAMC1-linked AKT, ERK, stromal, or metastatic phenotypes, but they cannot establish an osteosarcoma mechanism (Han et al., 2021; Ye et al., 2019; Fang et al., 2021; Fang et al., 2022).

Figure 2 therefore separates the single current osteosarcoma observation from five required tests: γ1-containing laminin assembly, extracellular deposition, defined receptor engagement, state-specific signaling, and phenotype with genetic perturbation and rescue. The ladder is evidentiary rather than a claimed linear pathway.

### 6.3 Experimental criteria for a functional dependency

A functional LAMC1 dependency requires genetic depletion or editing, biochemical and spatial confirmation of the relevant γ1-containing laminin, identification of the receptor and cellular compartment, and a state-specific phenotype rescued by LAMC1 re-expression or the appropriate assembled laminin. Laminin-binding receptors such as α3β1, α6β1, α7β1, and α6β4 must be assigned in the relevant isoform and structural context (Hohenester and Yurchenco, 2013). Although β4 Tyr1494-dependent signaling contributes to experimental lung colonization, the β4 study did not identify a laminin ligand or test LAMC1 (Wan et al., 2009). Failure of LAMC1 perturbation to alter deposition, receptor engagement, anoikis, re-adhesion, drug response, or pulmonary colonization would appropriately downgrade LAMC1 to a correlated marker.

## 7 Therapeutic vulnerabilities of matrix-state switching

Figure 4 integrates the translational and experimental implications of the framework. Panel A maps candidate intervention windows across the primary matrix-conditioned state, the detachment/anoikis bottleneck, and the pulmonary niche-adapted state. Panel B shows a linked transition assay in which the same population is followed from a defined bone-like matrix through poly-HEMA or ultra-low-attachment suspension and into a defined lung-like matrix with or without fibroblasts, with ligand/deposition, receptor activation, signaling, phenotype, and perturbation–rescue measured across the sequence. Lineage tracing or genetic barcoding is included to distinguish adaptation from clonal selection, while SAA is retained only as an exploratory pharmacological probe requiring osteosarcoma-specific validation.

**FIGURE 4:**
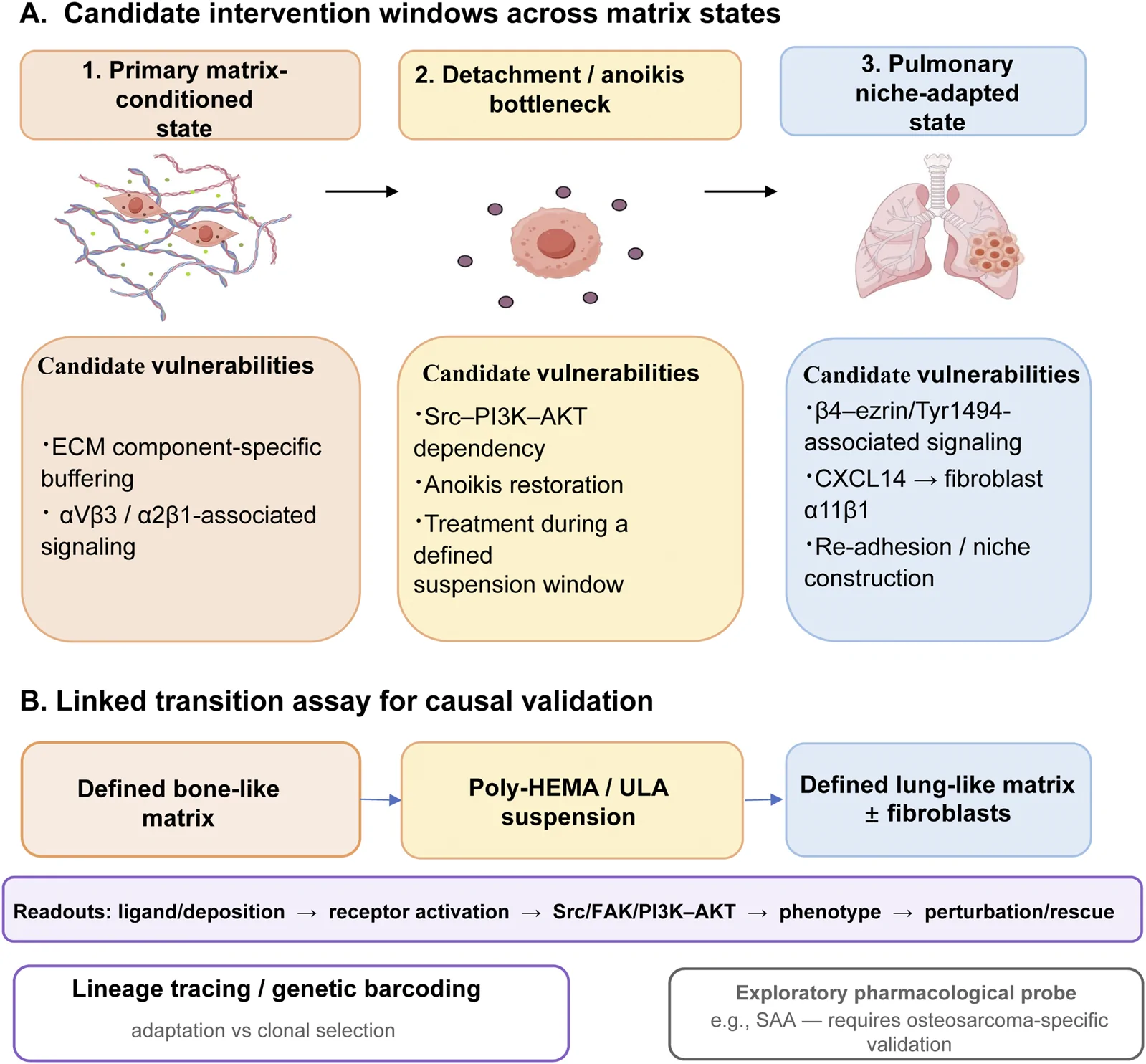
State-specific intervention windows and a linked experimental strategy for testing matrix-state switching. **(A)** Candidate intervention windows are mapped across the primary matrix-conditioned state, the detachment/anoikis bottleneck, and the pulmonary niche-adapted state. These vulnerabilities are hypothesis-generating rather than clinically established. **(B)** A linked transition assay follows the same osteosarcoma population from a defined bone-like matrix through poly-HEMA or ultra-low-attachment suspension and into a defined lung-like matrix with or without fibroblasts. Core readouts include ligand assembly/deposition, receptor abundance/activation, Src/FAK/PI3K–AKT or context-appropriate signaling, and state-relevant phenotypes, followed by perturbation–rescue. Genetic barcoding or lineage tracing can distinguish adaptation from clonal selection. SAA is shown only as an example of an exploratory pharmacological probe that requires osteosarcoma-specific validation.

### 7.1 One integrin is unlikely to control every disease state

Integrin dependencies vary by anatomical stage and cellular compartment. α4 can modify anoikis in suspended SAOS-2 cells in an epitope-dependent manner (Marco et al., 2003). β4 is disproportionately important for experimental pulmonary colonization (Wan et al., 2009). α11β1 operates in fibroblasts within the metastatic niche rather than serving as a simple malignant-cell receptor (Xu et al., 2024). A universal anti-integrin strategy would therefore ignore the very rewiring that accompanies progression.

Therapeutic vulnerability should be defined by disease state, compartment, and signaling output. A receptor that is dispensable in stable monolayer culture may become necessary during re-adhesion. A receptor abundant in bulk tissue may belong to stromal cells. The target of interest is the receptor-ligand architecture that becomes necessary at a particular transition.

### 7.2 The detached Src-PI3K-AKT state is a transient pharmacological bottleneck

Among the available states, detachment provides the sharpest mechanistic bottleneck. Anoikis-resistant cells maintained Src-dependent AKT activity after focal-adhesion signaling was lost, and inhibition of Src or PI3K restored cell death (Diaz-Montero et al., 2006). FAK inhibition alone would not be expected to collapse this state once survival has shifted to a FAK-independent topology. The relevant question is whether treatment can destabilize the Src-dependent suspended state without requiring prolonged systemic inhibition of Src-family signaling.

This temporal framing also helps interpret apparently inconsistent Src data. A pathway may be critical in a selected suspension model yet less predictive in another *in vivo* setting if the target window has passed or if alternative receptors have already been established. Matrix-state experiments should therefore specify when the inhibitor is applied relative to detachment and re-adhesion.

### 7.3 Re-adhesion and niche construction create a second intervention window

β4 perturbation and CXCL14-α11β1 blockade show that the pulmonary state contains targetable dependencies at both tumor and stromal levels (Xu et al., 2024; Wan et al., 2009). In the β4 model, suppression of the receptor or its Tyr1494-dependent signaling impaired metastatic colonization far more than primary tumor growth (Wan et al., 2009). In the CXCL14 model, blocking tumor-to-fibroblast signaling reduced TGF-β production, improved CD8-positive T-cell activity, and suppressed lung metastasis (Xu et al., 2024). A matrix-directed intervention can therefore act by preventing receptor reassembly or by stopping stromal construction of a permissive niche.

## 8 Experimental models that can test matrix-state switching

### 8.1 Transition models are more informative than static complexity alone

A three-dimensional culture is not automatically a matrix-state model. If composition, stiffness, oxygen, growth factors, and cellular compartments change at the same time, a phenotype may become more realistic while becoming less mechanistically assignable. Testing matrix-state switching requires the same tumor population to be followed through experimentally separable conditions of attachment, detachment, and re-adhesion, with the matrix variable defined at each step.

Existing osteosarcoma models already show why this matters. Collagen-based bioprinting reproduces invasive behavior and drug-response differences that are not apparent in flat culture (Pellegrini et al., 2022). Demineralized bone matrix produces a large shift in doxorubicin sensitivity and transcriptional state (Ren et al., 2024). Patient-derived organoids embedded into lung-derived matrix behave differently from the same organoids in collagen-rich gelatin matrices (Hipwood et al., 2026). The next step is to link such systems sequentially rather than using them as unrelated endpoints. Additional platforms now include immune/ECM-reconstituted 3D models, side-by-side scaffold comparisons, patient-derived tumoroids, an *in-vitro* hyaluronic-acid/GelMA/collagen bioprinted model, clinically correlated patient-derived organoids, and vascularized human-bone/CAM constructs (Pierrevelcin et al., 2022; Monette et al., 2025; Bhatia et al., 2026; Zhou et al., 2025; Sandhu et al., 2024; Nie et al., 2026; Smith et al., 2024).

### 8.2 Suspension systems remain indispensable for isolating anoikis

Poly-HEMA and ultra-low-attachment models strip away the matrix and therefore isolate loss of anchorage more cleanly than embedded 3D cultures. The SAOS-2 studies used this reductionist advantage to distinguish mitochondrial anoikis from FAK-independent Src-PI3K-AKT survival (Marco et al., 2003; Diaz-Montero et al., 2006). Their main limitation is that the experiment generally stops at the suspended endpoint.

A state-transition assay would recover viable cells after a defined suspension interval and transfer them into a controlled bone-like or lung-like matrix. Ligand state, integrin repertoire, Src/FAK/AKT activation, apoptosis threshold, and drug response could then be compared in the same lineage before detachment, during suspension, and after re-adhesion. For LAMC1, this design could distinguish a role in the attached state from a role that only emerges during metastatic re-entry into laminin-containing matrix.

### 8.3 Endogenous-matrix spheroids separate matrix production from imposed scaffold composition

Long-term osteosarcoma spheroids provide a setting in which tumor and stromal cells generate their own ECM. Cortini and colleagues showed deposition of type I collagen, type III collagen, and fibronectin at tumor-stromal interfaces; IL-6 neutralization reduced matrix deposition, while type I collagen reduced the cytotoxic effect of doxorubicin (Cortini et al., 2023). A 2024 spheroid study further showed that endogenous matrix production and oxygen diffusion interact to regulate chemotherapeutic response (Sagheb et al., 2024). Increased ECM production did not create a uniform resistance phenotype across all tumor conditions.

These findings support a distinction between matrix quantity and matrix state. Spheroids are well suited to spatial analysis of matrix deposition, hypoxia, receptor localization, and p-AKT, provided that central and invasive compartments are not pooled into one readout. They are less suited to assigning causality to one ligand unless matrix production is selectively perturbed.

### 8.4 Bone-derived scaffolds expose adhesion-metabolic coupling

Ren and colleagues reported that demineralized bone-matrix scaffolds increased doxorubicin tolerance in MG-63 and MNNG/HOS cells relative to 2D culture and enriched programs related to apoptosis control, drug transport, stemness, glutamine metabolism, focal adhesion, ECM organization, and matrix binding (Ren et al., 2024). These results support an adhesion–metabolic phenotype, but the multicomponent scaffold does not identify which matrix cue is causal.

Glutamine metabolism was functionally linked to the phenotype: inhibition reduced drug resistance, and α-ketoglutarate supplementation restored it (Ren et al., 2024). Matrix adaptation therefore extends beyond receptor-proximal signaling. A matrix state can create metabolic dependencies that may be therapeutically exploitable and may influence the activity of compounds evaluated only in monolayer culture.

### 8.5 Engineered matrices can uncouple biochemical identity from mechanics

Defined hydrogels provide a complementary advantage to tissue-derived scaffolds because composition and mechanics can be manipulated independently. A collagen-based bioprinted model supported viability, invasion, matrix degradation, and cisplatin-response studies in U-2 OS cells and a cisplatin-resistant derivative (Pellegrini et al., 2022). In 2D, the resistant U-2OS/CDDP 1 μg population showed only 14% Ki-67 positivity, whereas Ki-67 positivity increased to approximately 75% in 3D culture. Cells generated expanding clusters and matrix lacunae, accompanied by MMP-related remodeling (Pellegrini et al., 2022).

Collagen-only systems cannot directly test a laminin-γ1 hypothesis. The 2026 patient-derived osteosarcoma platform offers a closer fit (Hipwood et al., 2026). Matrix-free organoids were embedded into either collagen-rich GelMA or lung-derived decellularized ECM methacrylate (LungMA), with stiffness independently tuned to approximately 1 kPa or 4 kPa. Proteomic characterization showed that gelatin was predominantly collagen-based and lacked laminins, whereas lung dECM contained laminins and a broader matrisome (Hipwood et al., 2026).

Embedding converted compact organoids into invasive structures and increased doxorubicin resistance relative to both 2D cultures and matrix-free organoids. LungMA promoted more aggressive early invasion and greater chemoresistance than GelMA, particularly under the stiffer condition (Hipwood et al., 2026). The study used material from one patient donor and did not isolate which individual lung-matrix proteins generated the phenotype. The authors explicitly identified laminin-neutralization as a future experiment (Hipwood et al., 2026). This platform therefore provides a direct route for testing, rather than assuming, the laminin paradox.

### 8.6 Patient-derived organoids preserve tumor heterogeneity but do not remove matrix bias

Patient derivation does not by itself solve the ECM problem. Many organoid workflows rely on Matrigel or other basement-membrane extracts whose high laminin content supports growth but whose composition, growth-factor content, batch variability, and mechanical properties complicate ligand-specific interpretation. The 2026 PDOS study emphasized that matrix stiffness and composition should be controlled independently when the question is mechanobiological (Hipwood et al., 2026). Patient-derived cells grown in an undefined non-patient matrix preserve aspects of tumor heterogeneity while imposing an artificial ECM state. Recent patient-derived and scaffold-comparison studies make this limitation explicit: matrix choice alters osteosarcoma phenotype and drug response, and PDO-based chemosensitivity can correlate with clinical treatment response even when the culture matrix itself remains an experimental variable (Monette et al., 2025; Bhatia et al., 2026; Nie et al., 2026).

For translational drug screening, such matrices can still be useful. For causal studies of LAMC1 or integrin rewiring, a defined or tissue-derived matrix whose composition and mechanics are measured is preferable. The analytical goal is not maximal biomimicry at every step; it is sufficient biological relevance without losing the ability to assign cause.

### 8.7 Organ-on-chip systems can introduce the missing variable of sequential exposure under flow

Most 3D models hold cells in one matrix throughout the experiment. Osteosarcoma-on-chip technology begins to address this limitation. Lu and colleagues developed a decellularized osteosarcoma ECM-based micro-osteosarcoma and incorporated it into a recirculating microfluidic platform designed to reproduce bone-marrow niches, cell-cell interactions, cell-matrix interactions, and circulation (Lu et al., 2024).

For matrix-state switching, the value of such a system lies in its ability to create sequential compartments. A future device could connect a bone-like matrix, a low-adhesion perfusion interval, and a lung-derived matrix so that one lineage experiences primary attachment, dissemination-like detachment, and re-adhesion under controlled flow. This would capture the transition itself rather than comparing unrelated static cultures.

### 8.8 A minimum falsification framework

A technically modest experiment can already test the central hypothesis. The same osteosarcoma population can be studied in three sequential states: attached on a defined primary-site matrix, detached on poly-HEMA or ultra-low-attachment culture, and re-adhered into a defined lung-like matrix. Four layers should be measured at each transition: ligand assembly and deposition; receptor abundance and activation; FAK, Src, PI3K-AKT and context-appropriate downstream signaling; and phenotype, including anoikis, re-adhesion efficiency, invasion, drug response, and recolonization.

A strong LAMC1 experiment would add genetic depletion, measurement of γ1-containing laminin assembly and extracellular deposition, identification of the affected receptor, state-specific signaling, and rescue by LAMC1 re-expression or the relevant laminin matrix. An exploratory compound such as salvianolic acid A (SAA) should be considered only after this state sequence is defined. Chemoproteomic evidence supports covalent Raptor Cys65 engagement and mTORC1 inhibition outside osteosarcoma (Zheng et al., 2023), but no direct evidence links SAA to osteosarcoma matrix-state switching or LAMC1. Its role in this framework is therefore limited to future comparative testing across defined attachment, suspension, and re-adhesion conditions rather than therapeutic inference.

### 8.9 Model fidelity should be judged by mechanistic discriminability

No single model captures the full disease. Suspension culture isolates anoikis but removes matrix complexity. Spheroids generate endogenous ECM but confound it with oxygen and nutrient gradients. Bone-derived scaffolds preserve tissue cues but contain multiple inseparable signals. Defined hydrogels offer control at the cost of simplification. Patient-derived organoids preserve interpatient biology but remain sensitive to the matrix in which they are propagated. Microfluidic systems introduce flow and compartmental transitions but are technically demanding. Current 3D-model surveys and newer engineered systems similarly show that model choice changes the balance between mechanistic control, stromal complexity, native bone architecture, and throughput (Pierrevelcin et al., 2022; Zhou et al., 2025; Sandhu et al., 2024; Smith et al., 2024).

A linked hierarchy is more informative than one maximally complex model: a reductionist detachment assay to establish anoikis dependency; a defined re-adhesion matrix to identify ligand-receptor causality; a patient-derived or tissue-derived 3D system to test biological relevance; and an orthotopic or spontaneous metastasis model to validate the integrated phenotype. Complexity then serves as validation rather than a substitute for causal resolution.

## 9 Discussion and future directions

### 9.1 Where is the switch?

The evidence reviewed here supports a dynamic rather than static interpretation of osteosarcoma ECM biology. A matrix-state switch occurs when ligand presentation, receptor use, signaling topology, or the participating cellular compartment changes sufficiently to create a new survival architecture. The switch will not necessarily be visible as a single gene turning on or off. A receptor can remain expressed while changing ligand or adaptor partners; a kinase can remain phosphorylated while its upstream control changes; a matrix transcript can rise without producing a signaling-competent extracellular structure.

Future studies therefore need paired and spatially resolved measurements. Primary biopsies, invasive fronts, post-chemotherapy resections, and pulmonary metastases should be compared with spatial proteomics, matrisome profiling, multiplex imaging, and receptor-activation measurements rather than bulk RNA alone. Mature metastases are end points of selection and may not retain the transient receptor state that permitted initial colonization. Temporal sampling in experimental models is needed to locate the transition before it is obscured by later adaptation. This priority is consistent with modern ECM and integrin frameworks that emphasize spatially resolved matrix architecture and state-dependent mechanotransduction (Hynes and Naba, 2012; Mouw et al., 2014; Bonnans et al., 2014; Kechagia et al., 2019; Hamidi and Ivaska, 2018; Desgrosellier and Cheresh, 2010; Bergonzini et al., 2022; Jiang et al., 2022; Yuan et al., 2023; Su et al., 2020).

### 9.2 What is the bottleneck?

Detachment is currently the best-defined state-specific bottleneck, but the direct Src–PI3K–AKT evidence derives from a particular suspension-selected SAOSar model and should not be generalized to all osteosarcomas (Diaz-Montero et al., 2006). Later studies identify parallel, non-equivalent adaptations: FASN–ERK/Bcl-xL signaling (Sun et al., 2019), ID1–PI3K–AKT regulation (Zhao et al., 2019), FOSL1–SOX2-dependent stem-like properties (Wang et al., 2024), and Src–MAPK-associated angiogenesis (Gao et al., 2019). These observations nominate testable routes around the bottleneck rather than one universal anoikis program.

The clinically useful question is therefore not simply which pathway is active in a metastasis. It is which transition is nonredundant enough that failure to complete it eliminates metastatic fitness. That distinction will determine whether a target should be inhibited continuously, during a short dissemination window, or after cells enter the pulmonary niche.

### 9.3 When does LAMC1 become functional?

LAMC1 provides a stringent test of the framework because the current evidence stops at the transcriptomic-candidate level in osteosarcoma (Tang et al., 2022). Functional relevance begins only when γ1 protein is incorporated into a defined laminin heterotrimer, deposited extracellularly, engaged by a compatible receptor, and linked causally to a state-specific phenotype. The contrast between laminin-poor GelMA and laminin-containing LungMA now offers an experimentally controlled setting in which this sequence can be tested (Hipwood et al., 2026). Structural and cross-cancer laminin evidence defines the intervening biochemical steps that must be resolved before such a pathway can be assigned (Hamill et al., 2009; Hohenester, 2019; Ido et al., 2007; Pulido et al., 2017; Arimori et al., 2021; Nonnast et al., 2025; Zhang et al., 2017; Kunitomi et al., 2020; Fang et al., 2022; Bai et al., 2024; Liu et al., 2019; Bai et al., 2022).

LAMC1 depletion, laminin-neutralizing strategies, receptor blockade, and rescue should be applied across attached, detached, and re-adhered states. A phenotype that emerges only in lung-like matrix would support the interpretation of LAMC1 as a state-dependent matrix dependency. Failure to alter deposition, receptor engagement, or phenotype would appropriately downgrade LAMC1 to a correlated marker. Either result is more informative than inferring a LAMC1-AKT pathway from coexpression.

### 9.4 How should matrix-aware therapy be tested?

Most anticancer screens are performed in stable adherent monolayers. Such assays are poorly suited to detecting drugs that preferentially kill detached cells, prevent re-adhesion, disrupt fibroblast-mediated niche construction, or reverse matrix-conditioned resistance. Matrix-aware pharmacology should therefore compare the same agent before detachment, during suspension, and after re-adhesion, while keeping exposure timing explicit.

Readouts should also extend beyond one metabolic viability assay. The 2026 patient-derived organoid study showed divergence between viability-based measures and functional invasion inhibition under chemotherapy (Hipwood et al., 2026). Anoikis, re-adhesion efficiency, invasion, matrix remodeling, and metastatic outgrowth should therefore be measured alongside cytotoxicity. Exploratory pharmacological probes should be interpreted only after their state selectivity has been established prospectively, rather than because they modulate the same phosphoprotein across unrelated tumor models.

### 9.5 Clinical translation requires paired states, not only larger cohorts

Large transcriptomic cohorts can nominate matrix-associated markers but cannot establish switching on their own. The most informative clinical series would pair pretreatment primary biopsy, post-chemotherapy resection, pulmonary metastasis, and recurrent or resistant tissue when available. A clinically useful biomarker may be the change in a matrix module between states rather than its absolute abundance at one time point.

For LAMC1, evidence would be much stronger if increased γ1-containing laminin deposition at the pulmonary site were accompanied by acquisition of a compatible receptor and state-specific signaling. For the stromal arm, paired measurements of fibroblast α11β1, TGF-β activity, matrix contractility, and immune exclusion could identify a metastatic niche state that would be invisible in malignant-cell-only assays. These designs also provide a route to validate the small clinical FAK dataset (Le Guellec et al., 2013) and to determine whether receptor recovery, as observed for β4 in experimental metastases (Wan et al., 2009), occurs in patients. A recent single-cell and spatial atlas provides a clinically relevant foundation for this paired, compartment-resolved strategy (Zheng et al., 2024a).

### 9.6 Conclusion

Osteosarcoma progression is not well represented by a fixed ECM-integrin-FAK-AKT pathway. The available evidence instead describes recurrent reorganization of ligand-receptor relationships as cells move through primary growth, detachment, anoikis escape, and pulmonary colonization. Integrin rewiring is a measurable mechanism of that change. Anoikis escape is a major biological bottleneck, and pulmonary re-adhesion creates a second opportunity for intervention. Matrix composition, mechanics, metabolism, and stromal participation determine whether these transitions succeed.

LAMC1 remains a hypothesis-generating candidate with a clearly defined evidentiary boundary. Demonstrating state-specific laminin assembly, receptor engagement, signaling, and rescue would elevate it from a transcriptomic association to a functional matrix dependency; failure to establish those steps should limit the claim to correlation. Future *in vivo* studies should also distinguish true same-clone state transitions from selective expansion of pre-existing populations. Stable genetic barcoding, lineage-resolved paired sampling, and state-responsive dual-reporter systems that remain traceable after matrix digestion could determine whether individual osteosarcoma lineages traverse multiple matrix states or whether distinct subclones preferentially occupy them.

The central therapeutic opportunity may therefore lie not in permanently disabling one extracellular-matrix pathway, but in preventing osteosarcoma cells from successfully switching from one matrix-dependent survival state to the next.
